# Introducing non-cognitive load to the educational discourse

**DOI:** 10.3389/fpsyg.2024.1411102

**Published:** 2025-01-13

**Authors:** Gulbakhyt Sultanova

**Affiliations:** Center for Pedagogical Measurements, Nazarbayev Intellectual Schools, Astana, Kazakhstan

**Keywords:** non-cognitive load, non-cognitive skills, cognitive load theory, theoretical framework, holistic student development, academic achievement

## 1 Introduction

In the dynamic landscape of education, the pursuit of holistic student development has long been associated with a dual emphasis on cognitive and non-cognitive skills (Boman, 2023). While cognitive skills traditionally dominate discussions on educational outcomes (Roth et al., 2015), the recognition of non-cognitive skills as equally pivotal components in shaping a student's overall growth has gained prominence (Napolitano et al., 2021). Referred to as “generic competences,” “life skills,” “21st-century skills,” and similar terms (Cinque et al., 2021), these skills go beyond conventional cognitive abilities. They include a varied spectrum of personal attributes, social skills, and character traits that impact an individual's capacity to learn and excel in academic settings (Jones et al., 2019; Lechner et al., 2019).

The cultivation of both cognitive and non-cognitive skills places a certain burden on students. In alignment with the established concept of cognitive load (Sweller, 2011), a parallel consideration should be given to non-cognitive load, an uncharted aspect that holds the potential to either facilitate or hinder the advancement of non-cognitive skills. Recent studies indicate that non-cognitive aspects, such as self-efficacy, emotional regulation and motivation (Napolitano et al., 2021; Hartelt and Martens, 2024; Zhang, 2024), significantly impact cognitive load by influencing resource allocation and engagement, highlighting the interplay between these dimensions in learning processes. This opinion article aims to introduce the novel concept of non-cognitive load, paving the way for its instrumentalization in the development of a robust theoretical framework and practice-oriented assessment tools. By acknowledging the role of non-cognitive load, the discussion embarks on a journey to enhance understanding of the nuanced elements shaping the holistic educational experience.

## 2 Defining non-cognitive load

Educational research has long centered around cognitive processes, with Cognitive Load Theory being a prominent framework (Sweller, 2011; Sweller et al., 2019). This theory explores the cognitive demands placed on learners during the acquisition of new knowledge, highlighting the finite capacity of working memory and the importance of optimizing instructional designs for effective cognitive load management. The theory recognizes three types of cognitive load: intrinsic, extraneous, and germane (Paas et al., 2003; Sweller, 2011; Zheng, 2017). Intrinsic cognitive load pertains to the inherent difficulty of learning materials or tasks, with some topics carrying higher complexity. Extraneous cognitive load involves unnecessary cognitive processing demands imposed by instructional design or the learning environment, potentially arising from poorly designed materials. Germane cognitive load, whose independent status in Cognitive Load Theory has been debated (Sweller, 2023), concerns mental effort directed at organizing and integrating information into long-term memory, beneficial for learning and problem-solving.

As Sweller et al. (2019) summarized the evolution of Cognitive Load Theory from 1998 to 2018 and offered insights into potential avenues for future research, they advocated for a nuanced approach that recognizes the role of emotions, stress, and uncertainty in learning. These elements have already been identified as potential factors that constrain working memory capacity, elevate cognitive load, and hinder learning and transfer (Finell et al., 2022; Pellizzoni et al., 2022). Building on this, a specific load emerges linked to the development of non-cognitive skills, addressing emotional regulation, social dynamics, and motivation. This is termed non-cognitive load.

This load, much like its cognitive counterpart, can either facilitate or hinder the development of non-cognitive skills. When students face high levels of emotional stress, social pressure, or motivational challenges, their cognitive resources may diminish, hindering productive learning (Moran, 2016). Effectively managed non-cognitive load may positively enhance emotional intelligence, strengthen social connections, and foster resilience. Conversely, an imbalanced non-cognitive load may lead to stress, emotional exhaustion, and diminished motivation, impeding the development of these crucial skills.

The concept of non-cognitive load distinguishes itself from both the affective domain in the Taxonomy of Educational Objectives known as Bloom's Taxonomy (Krathwohl et al., 1964) and cognitive load in Cognitive Load Theory (Sweller, 2011) by its focus, structure, and application (Table 1). Unlike Bloom's affective domain, which emphasizes hierarchical emotional and attitudinal development over time, non-cognitive load addresses real-time emotional, social, and motivational demands that directly influence learning processes. In contrast to cognitive load, which targets the mental effort required for task-specific cognitive processing, non-cognitive load encompasses broader challenges, such as managing emotions, navigating social interactions, and sustaining motivation. Structurally, non-cognitive load parallels intrinsic, extraneous, and germane dimensions of Cognitive Load Theory but applies these to cultivating emotional resilience, fostering teamwork, and supporting overall engagement. It bridges immediate classroom realities with broader developmental goals, positioning non-cognitive load as a dynamic and multidimensional framework that complements traditional educational practices.

**Table 1:** Comparison of affective domain, cognitive load, and non-cognitive load.

**Aspect**	**Affective domain (taxonomy of educational objectives)**	**Cognitive load (cognitive load theory)**	**Non-cognitive load**
Focus	Emotional and attitudinal aspects of learning (values, attitudes, and emotions)	Cognitive processing demands (memory, problem-solving)	Emotional, social, and motivational demands impacting learning processes
Structure	Hierarchical: receiving → responding → valuing → organizing → internalizing values	Multidimensional: intrinsic, extraneous, and germane cognitive loads	Multidimensional: inherent, unnecessary, and beneficial non-cognitive loads
Domain of impact	Long-term emotional and attitudinal development in learning	Cognitive processing efficiency during learning activities	Dynamic factors affecting emotional, social, and motivational states in real time
Application	Fostering values and attitudes over time (e.g., promoting empathy, ethical reasoning)	Designing instructional materials for optimal cognitive efficiency	Managing and optimizing emotional, social and motivational demands to enhance learning
Measurement	Qualitative assessment of attitudes, values, and emotional responses	Quantitative measures of cognitive effort (e.g., task difficulty, working memory load)	Potential for quantitative measurement using surveys and real-time analytics (e.g., stress or motivation indices)
Interventions	Activities to develop empathy, ethical reasoning, and emotional intelligence	Simplifying materials to reduce extraneous load or building schemas to enhance germane cognitive load	Reducing unnecessary non-cognitive load (e.g., minimizing classroom stress) or enhancing beneficial non-cognitive load (e.g., fostering teamwork)
Temporal scope	Long-term development of affective attributes	Primarily short-term, focusing on task-specific cognitive processing	Short-term and long-term interplay between emotional, social, and motivational factors and learning
Key contributions	Emphasizing the importance of emotional and value-based learning goals	Highlighting the importance of cognitive efficiency and task complexity in learning	Integrating emotional, social, and motivational dimensions into learning frameworks to bridge cognitive and affective domains
Criticism	Limited direct application to real-time classroom dynamics	Overemphasis on cognitive processes, neglecting emotional and social aspects	Novel and still under theoretical development; requires empirical validation

## 3 Types of non-cognitive load

**Figure 1:**
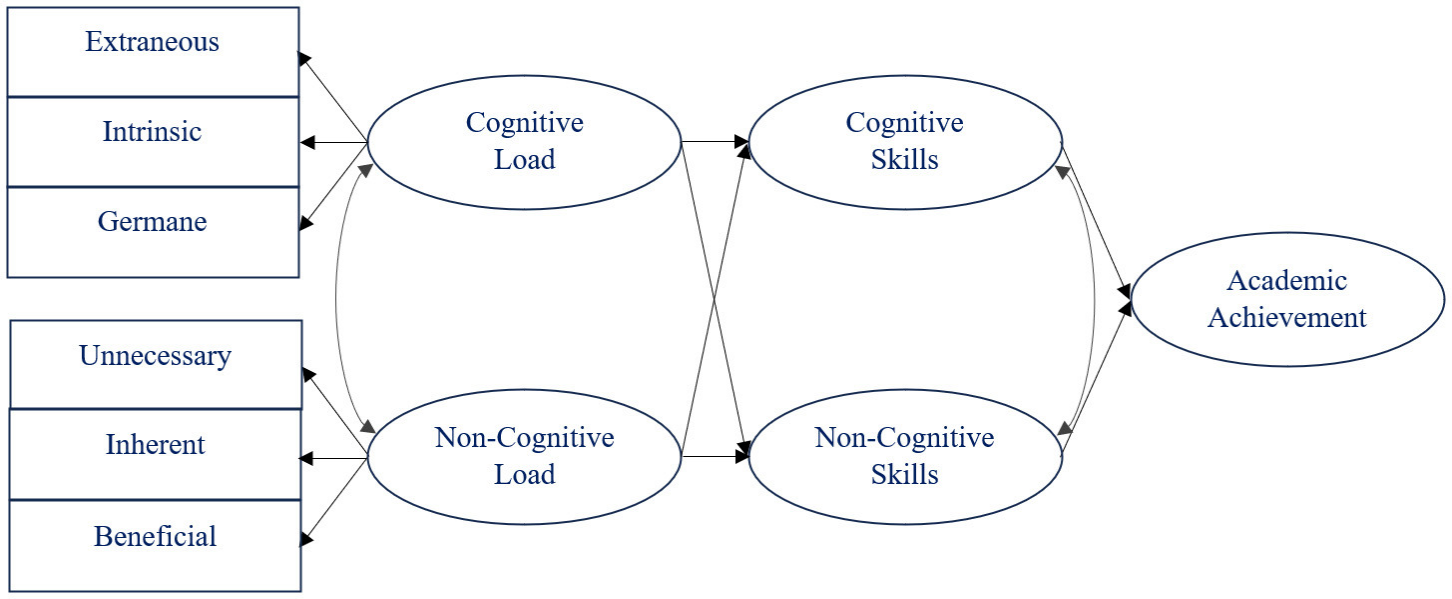
Theoretical framework.

Drawing from Cognitive Load Theory (Sweller, 2011; Sweller et al., 2019), three types of non-cognitive load can be delineated—unnecessary (extraneous), inherent (intrinsic), and beneficial (germane)—recognizing the interconnectedness of cognitive and non-cognitive functions in learning (Figure 1).

In line with extraneous cognitive load, unnecessary non-cognitive load refers to distractions or irrelevant factors that impede learning tasks. Students may encounter this type of non-cognitive load in environments where disruptive stress or anxiety impedes their ability to concentrate on the actual learning content. Examples of unnecessary non-cognitive load include excessive worry or anxiety about social situations, destructive emotional reactions that disrupt concentration, or irrelevant motivational factors that detract from the task at hand. Similar to extraneous cognitive load, unnecessary non-cognitive load hampers optimal performance and can lead to suboptimal outcomes across various domains. Addressing this type of non-cognitive load enables educators and individuals to create environments that reduce unnecessary distractions, improve emotional regulation, encourage positive social interactions, and foster sustained motivation.

Reflecting intrinsic cognitive load, inherent non-cognitive load encompasses natural or essential elements of non-cognitive load related to emotional, social, and motivational complexities. When engaged in a collaborative project, students may encounter this type of non-cognitive load as they manage emotions related to teamwork, navigate social dynamics, and sustain motivation throughout the project. For example, inherent non-cognitive load tasks may include recognizing and labeling emotions, establishing social connections, setting goals, and maintaining self-discipline. These tasks represent the core building blocks of non-cognitive skills and are crucial for individuals to navigate everyday challenges and achieve personal and academic goals. Similar to intrinsic cognitive load, inherent non-cognitive load is an integral part of the learning process and encompasses the foundational emotional, social, and motivational challenges associated with the learning content or context. This type of non-cognitive load contributes significantly to fostering resilience, adaptability, and interpersonal skills, which are increasingly valued in today's complex and interconnected world.

Corresponding to germane cognitive load, beneficial non-cognitive load pertains to enriching or constructive aspects of non-cognitive load that facilitate positive processing of emotional, social, and motivational dimensions. Examples include reflecting on and making sense of one's emotions in a learning context or intentionally strategizing to enhance motivation. In educational settings, this type of non-cognitive load encompasses tasks and activities that directly contribute to the development of non-cognitive skills. For example, engaging in reflective practices to understand and manage one's emotions effectively or participating in collaborative activities to enhance social interaction skills can be considered beneficial non-cognitive load tasks. Similar to germane cognitive load, beneficial non-cognitive load facilitates deeper learning and skill acquisition by focusing on meaningful engagement with non-cognitive elements. Understanding and intentionally engaging with this type of non-cognitive load can foster a more effective and positive learning experience, emphasizing the importance of addressing emotional, social and motivational dimensions for holistic student development.

## 4 Effects of non-cognitive load

The differentiation and understanding of three types of non-cognitive load can provide educators with valuable insights into the nuanced dynamics of students' emotional and social experiences as well as their motivation throughout the learning process. For this purpose, it is essential to explore the influence of non-cognitive load on the development of non-cognitive skills alongside investigating the impact of cognitive load on the advancement of cognitive skills (Figure 1). Similar to how cognitive load influences the enhancement of non-cognitive skills (Redifer et al., 2021), non-cognitive load may also modify the improvement of cognitive skills. Concurrently with cognitive load, non-cognitive load could play a role in shaping academic achievement by contributing to the comprehensive development of students' skills.

In this conceptualization, non-cognitive load becomes a counterpart to cognitive load, highlighting the holistic nature of the learning process that involves both cognitive and non-cognitive elements. This framing recognizes that effective learning involves the integration of cognitive and non-cognitive skills, and both contribute to the overall effort expended during learning activities (Boman, 2023). It is expected that reducing unnecessary non-cognitive load, optimizing inherent non-cognitive load, and increasing beneficial non-cognitive load can improve the development of cognitive and non-cognitive skills, thereby positively affecting students' academic achievement. To prevent non-cognitive burden, educators can focus on fostering emotional regulation, building self-efficacy through positive feedback, promoting intrinsic motivation, encouraging collaborative learning to support social interactions, developing resilience through gradual challenges, and ensuring task complexity is manageable, thus creating a supportive environment that enhances learning outcomes.

The role of non-cognitive load in shaping the development of non-cognitive skills provides insights into contentious findings regarding their influence on students' academic success. This is particularly evident in the case of grit and growth mindset, concepts extensively studied by experts and endorsed by practitioners. Credé (2018) highlights that critical claims about grit lack thorough examination or contradict empirical evidence, questioning its ability to predict educational success or respond effectively to interventions. Similarly, efforts to validate growth mindset theories have often fallen short of expected results (e.g., Bahník and Vranka, 2017). A recent meta-analysis by Macnamara and Burgoyne (2023) underscores the infrequent and potentially misleading positive outcomes associated with growth mindset interventions. Incorporating the notion of non-cognitive load into these discussions may provide clarity concerning its influence on developing non-cognitive skills and improving academic performance.

## 5 Measuring non-cognitive load

To effectively measure non-cognitive load, it is crucial to assess its three types through diverse methods. For unnecessary non-cognitive load, self-report scales focusing on emotional stress and task difficulty can help identify sources of irrelevant cognitive demand. Inherent non-cognitive load, linked to the task's complexity, can be measured through observational tools that assess students' engagement and task-related challenges. To measure beneficial non-cognitive load, educators can observe students' motivation levels, collaboration, and resilience, while physiological measures (e.g., heart rate variability) can provide objective data on emotional engagement. Combining these methods allows for targeted interventions that reduce unnecessary non-cognitive load, optimize inherent non-cognitive load, and enhance beneficial non-cognitive load, promoting both skill development and academic success.

## 6 Implications for education

From a theoretical perspective, integrating non-cognitive load into the learning process invites important questions about its impact on skill development and academic achievement (Figure 1). Understanding how excessive emotional or motivational demands, such as sustaining optimism and regulating energy (Sultanova and Shora, 2024; Sultanova et al., 2024), can influence resource allocation is crucial for unraveling the complex relationship between non-cognitive load and cognitive outcomes. Theoretically, this raises broader concerns about the balance between the types of non-cognitive load and their respective influence on student performance.

Practically, educators can leverage this understanding by identifying and managing non-cognitive stressors in the classroom. For instance, creating supportive environments that minimize emotional or social overwhelm—through flexible seating, engaging materials, and mindful use of technology—can enhance student engagement. Furthermore, student-centered approaches that encourage collaboration can reduce social burden, while real-world tasks can address motivational pressure. Tailoring teaching strategies to optimize non-cognitive factors helps foster a more balanced learning environment, ultimately promoting both cognitive and non-cognitive skill development.

## 7 Conclusion and future directions

This opinion article has set the stage for a more comprehensive understanding of education through the introduction of the concept of non-cognitive load. By broadening the cognitive load model to include emotional, social, and motivational dimensions, new pathways for advancing educational research have been illuminated. Reflecting on the preliminary dimensions and relationships within the conceptual framework underscores the importance of considering both cognitive and non-cognitive factors in pedagogical practices and policies. Looking forward, there is a call for further research, urging interdisciplinary collaboration and engagement with stakeholders to refine and validate the understanding of non-cognitive load in diverse educational contexts.
